# Clinical feasibility and benefits of a tapered, sand-blasted, and acid-etched surfaced tissue-level dental implant

**DOI:** 10.1186/s40729-020-00234-6

**Published:** 2020-08-06

**Authors:** Buyanbileg Sodnom-Ish, Mi Young Eo, Truc Thi Hoang Nguyen, Myung-Joo Kim, Soung Min Kim

**Affiliations:** 1 Department of Oral and Maxillofacial Surgery, Dental Research Institute, School of Dentistry, Seoul National University, 101 Daehak-ro, Jongno-gu, Seoul, 03080 Korea; 2 Department of Prosthodontics, Dental Research Institute, School of Dentistry, Seoul National University, Seoul, Korea

**Keywords:** Success rate, A tapered tissue-level implant, Sand-blasted, Acid-etched

## Abstract

**Abstract:**

**Background:**

It has been 50 years since Brånemark first introduced the concept of osseointegration. Since then, numerous ongoing research, developments, and optimization of implant properties have been conducted. Despite the high survival and success rates of dental implants, failures still occur in a small number of patients that are being rehabilitated by implants. The purpose of this study was to evaluate the survival and success rate of the Stella® implants that incorporate sand-blasted and acid-etched (S&E) surface treatment and tapered body design to confirm their clinical feasibility and benefits after placement.

**Methods:**

We reviewed 61 partially and fully edentulous patients who underwent a tapered, S&E surfaced tissue-level implant placement between May 2013 and February 2016 in the Department of Oral and Maxillofacial Surgery in the Seoul National University Dental Hospital. Patient characteristics and treatment results were collected, and records of dental implants were analyzed clinically and radiologically.

**Results:**

A total of 105 implant fixtures were placed in these patients. The mean age at the time of the surgery was 63.7 years with a range of 31 to 88 years. In total, 4.0-mm and 4.5-mm diameter implants were the most frequently used dental implants (40%, 49%) in this study. Implants 8.5 mm in length were predominantly used (60%). Seventy dental implants were placed in the mandible (70%), and only one dental implant was placed in the maxillary anterior region. At the end of the 5-year observation period, the success rate of the Stella® implants was 98.1%. Among the 105 implants placed, 2 were considered to be failures. Summarizing the clinical and radiographic results, the remaining 103 implants were considered successfully integrated.

**Conclusion:**

The overall success rate was 98.1%. The tapered, S&E surfaced tissue-level implant system exhibited great performance in a variety of clinical situations including failed implant sites that enabled predictable and successful treatment outcomes. The effectives of a tapered design of tissue level, not a parallel design, are shown in this clinical report.

## Background

It has been 50 years since Brånemark et al. first introduced the concept of osseointegration [1, 2]. Since then, numerous ongoing research, developments, and optimization of implant properties have been conducted. Dental implants are now available in various designs, lengths, diameters, implant surfaces, implant-abutment junctions, and platforms. Despite the high survival and success rates, implant failures still occur in a small number of patients that are being rehabilitated by implants [3].

Implant failures can be categorized as early (before abutment connection) and late (after implant loading) failure, depending on when they occur. Early failure is caused by the inability to establish an intimate bone-to-implant contact. The implant designs including the width, length, surface characteristics, thread design, and shape have been identified as one of the risk factors that contribute to early failure [4, 5]. Despite the numerous attempts made by various manufacturing companies to enhance osseointegration and prevent complications, there is no evidence to suggest the superiority of any specific type of implant [6, 7].

The Stella® implants (Stella, Shinhung Co., Seoul, Korea) have incorporated specific design features that enhance osseointegreation: a 1° tapered body with 2.5° tapered apical design, sand blasted and acid etched (S&E) surface, double thread with a thread of 35°, helix cutting edge, and an 8° conical seal design. Several reports have demonstrated the improvements to the primary stability with implants of sand blasted, large grit and acid-etched (SLA) surface-treated implants [8], specific cutting thread pattern [9], and with implants of tapered design compared with standard implants [10].

Alterations to the surface and the macro geometry and/or surgical instrumentations of the implant body have been shown to produce notable effects during the early stages of bone healing around the implants [11]. SLA implants that have a micro-roughened surface have exhibited better early osseointegration. S&E surface-treated implants stimulate cell differentiation and protein production, creating a large amount of bone-to-implant contact. This fact has been demonstrated in various animal studies. In a preclinical study by Buser et al., bone-to-implant contact with different surface modifications was studied. The bone-to-implant contact was 50–60% in S&E surface implants, whereas titanium plasma-sprayed implants exhibited 30–40% bone-to-implant contact [12]. In a 5-year multicenter prospective study by Cochran et al., SLA implants exhibited 99.1% and 98.8% survival and success rates, respectively, for 385 dental implants installed in 120 patients [13].

The purpose of this study was to evaluate the clinical feasibility and benefits of Stella® implants placed in various clinical cases. In this study, we analyzed the clinical and radiographic data findings following implant placement and loading. The influencing factors on marginal bone loss such as patient age, sex, systemic disease, location, distribution of the implants, and their diameters and lengths were also analyzed.

## Methods

This retrospective study is reported following Strengthening the Reporting of Observational studies in Epidemiology (STROBE) guidelines [14, 15]. From May 2013 and February 2015, 61 partially and fully edentulous patients who underwent Stella® implant placement between in the Department of Oral and Maxillofacial Surgery in the Seoul National University Dental Hospital were reviewed. The study protocol and access to patient records were approved by the Institutional Review Board of Seoul National University, Seoul, Korea (Institutional Review Board approval number S-D20200007). A total of 105 implants fixtures were placed, and all implants were placed according to the manufacturer’s surgical protocol by a single oral and maxillofacial surgeon.

### Inclusion criteria

We included medically compromised patients with osteoporosis and hypertension and oral cancer patients with ablation therapy, with or without radiotherapy (Table 1). Their clinical records and radiological findings were examined. Depending on the patient’s condition and treatment plan, the Stella® implants were placed through one or two-stage surgery ranging from 3 months to 2 years. An immediate panoramic radiograph was taken after implant placement. Implant prognosis was evaluated according to The International Congress of Oral Implantologists (ICOI) Pisa Consensus implant health scale [16].

**Table 1:** Details of medically compromised patients

No.	Age	Gender	Position of implant placement	Diameter of implant	Length of implant	Implant prognosis	Past medical history
1	68	F	#46i	4.0	8.5	Success	HTN, osteoporosis, DM
			#47i	4.0	8.5	Success	
2	63	F	#44i	4.0	10	Success	Lymphoma
			#45i	4.0	7	Satisfactory survival	
			#46i	4.0	7	Success	
3	73	M	#44i	5.0	10	Success	HTN, DM
4	68	M	#46i	4.5	10	Success	HTN, DM
			#47i	4.5	8.5	Success	
			#35i	4.0	10	Satisfactory survival	
			#36i	4.0	8.5	Success	
			#37i	4.0	7	Success	
5	69	F	#37i	4.5	8.5	Success	HTN, DM, osteoporosis
			#36i	5.0	10	Success	
6	72	F	#17i	4.0	7	Success	HTN
			#16i	4.0	8.5	Success	
			#26i	4.0	7	Success	
7	72	M	#36i	4.5	8.5	Success	HTN
			#37i	4.5	8.5	Satisfactory survival	
8	77	M	#45i	4.5	10	Success	HTN, DM, bleeding tendency
9	86	F	#37i	4.0	10	Success	HTN, bleeding tendency
10	74	F	#47i	4.5	10	Success	HTN
11	53	M	#47i	4.5	8.5	Success	HTN, bleeding tendency
			#14i	4.0	8.5	Success	
			#24i	4.0	8.5	Success	
			#25i	4.0	7	Success	
			#26i	4.0	7	Success	
12	75	F	#36i	4.0	8.5	Success	Osteoporosis, bisphosphonate usage
			#37i	4.0	8.5	Success	
			#25i	4.5	8.5	Success	
13	71	F	#46i	4.5	8.5	Success	HCV
14	31	M	#46i	4.0	8.5	Success	Mental disability
			#47i	4.0	8.5	Success	
15	70	F	#16i	4.5	8.5	Success	HTN, osteoporosis
16	76	M	#47i	4.5	7	Success	SCC
17	80	F	#45i	4.0	8.5	Satisfactory survival	HTN, osteoporosis
			#46i	5.0	8.5	Success	
18	58	F	#37i	4.0	8.5	Success	HTN, bleeding tendency
19	78	F	#17i	5.0	7.0	Failure	HTN, osteoporosis, hypothyroidism
			#47i	4.5	8.5	Success	
			#46i	5.0	8.5	Success	
20	39	M	#36i	4.5	7	Success	Tongue cancer
21	62	F	#46i	4.5	8.5	Success	Bullous pemphigoid
22	69	M	#26i	5.0	8.5	Success	HTN, osteoporosis, angina pectoris
23	76	F	#46ii	5.0	10	Satisfactory survival	DM, osteoporosis
24	75	F	#47i	4.5	7	Success	HTN, DM
			#36i	4.0	8.5	Success	
25	69	F	#16i	4.0	8.5	Success	HTN, osteoporosis
			#17i	4.0	8.5	Success	
26	46	M	#16i	4.0	10	Success	DM
27	82	F	#47i	4.5	8.5	Success	HTN, lung cancer, thyroid surgery
28	57	F	#16i	4	8.5	Compromised survival	Osteoporosis
			#17i	4	8.5	Success	
29	65	M	#36i	M	8.5	Success	HTN, hyperlipidemia, gout

*HTN* hypertension, *HCV* hepatitis C virus, *SCC* squamous cell carcinoma, *DM* diabetes mellitus

### Exclusion criteria

The exclusion criteria are as follows:

(1) patients who did not follow the follow-up

(2) patients who did not have the panoramic radiogram for evaluation.

### Implant types and site of placement

**Fig. 1:**
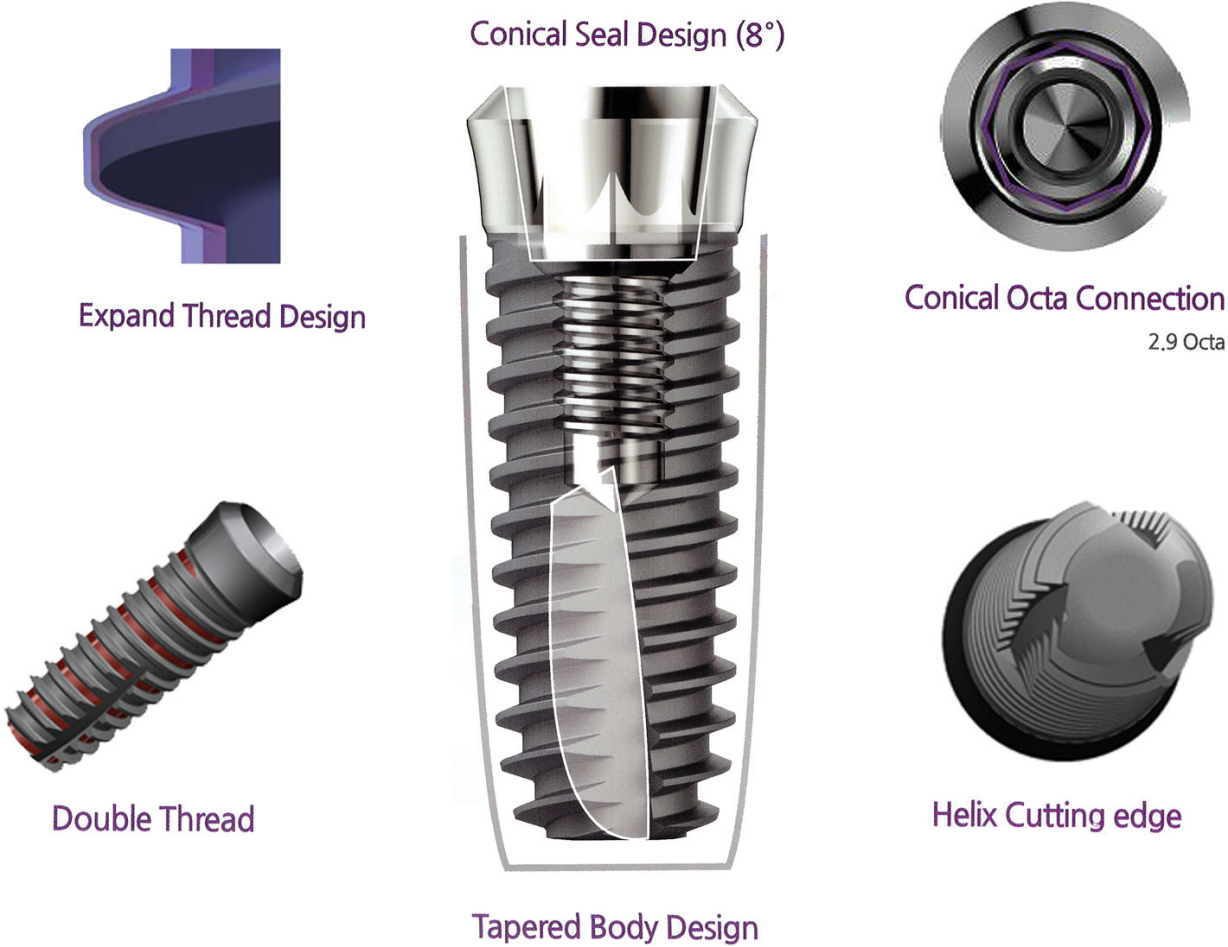
Macroscopic design of the Stella® implant system used in this study. The 8° conical seal design located in the prosthetic connection part prevents screw loosening. The three spiral cutting edges enable self-tapping, facilitate rapid implantation, and minimize the resistance of the bone

**Fig. 2:**
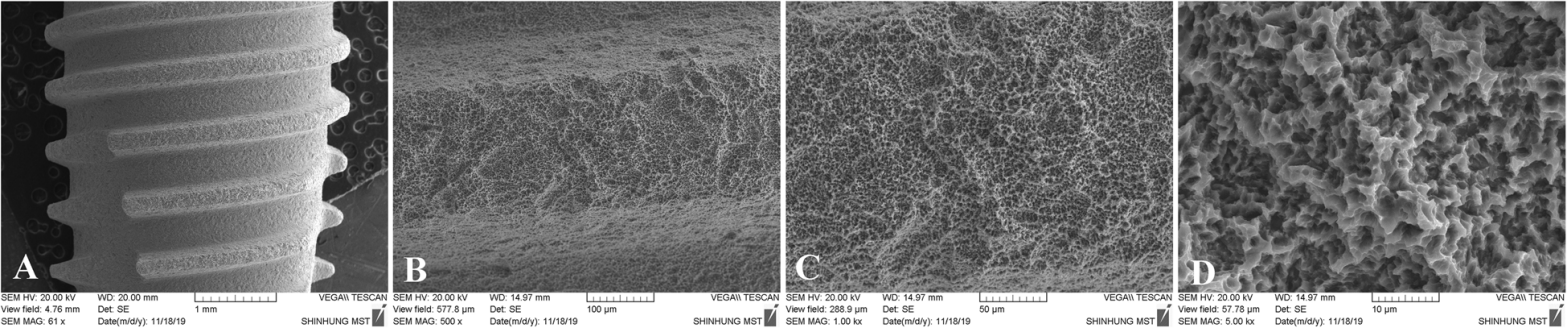
SEM view of the surface morphology of the tapered, sand-blasted, and acid-etched (S&E) surface. SEM × 60 magnification view (**a**), × 500 magnification view (**b**), × 1000 magnification view (**c**), and × 5000 magnification view (**d**)

The implant system used in this study was a one stage, non-submerged type with an 8° taper body that could obtain better primary stability with a higher torque value. The Stella® implant system incorporated the double thread design that reduced the placing time and improved the stability during initial penetration. The Octa 2.9 mm internal connection design can prevent rotational micromovements or micro gap enlargements. The helix cutting edges enable self-tapping, easy assembly, and minimal bone resistance by penetration. The double thread with a 35° spiral helix allows for rapid and firm implantation (Fig. 1). The S&E surface, which has been developed by adopting the SLA surface treatment method, has a surface roughness of Ra 2.5 um or more that increases the bone healing period and cell response by more than 20% (Fig. 2a–d). Furthermore, the Stella® implant system gained superior primary fixation even in soft bone.

For maxillary sinus grafting, ridge augmentation, and guided bone regeneration (GBR), an allograft obtained from cortical or cancellous bone, OraGraft® (LifeNet Health Inc., VA, USA), and autogenous bone graft harvested from the mandibular ramus were used.

### Surgical procedure

All of the surgical procedures were performed under local anesthesia using lidocaine with or without epinephrine. Prior to implant placement, a full thickness flap in the edentulous area was elevated following the manufacturer’s guidelines. An intraoral crestal incision was created followed by the mucoperiosteal dissection of the mucoperiosteum. All the implants were placed following the manufacturer’s surgery protocol. For sinus lifting procedures, a crestal and lateral approach was used. Sinus lifting using allogenic grafting materials was performed in 5 patients who received implants in the maxilla.

Seven implants were placed in sites with bone grafting, while ninety-eight implants were placed without bone grafting. Depending on the patient’s condition and treatment plan, the implant systems were placed through one or two-stage surgery ranging from 3 months to 2 years. Seventy-nine implants were placed using the one stage method, while 26 implants were placed using the two-stage stage method.

### Assessment and analysis

Patient data including age, sex, presence systemic disease, location of implant placement, and implant diameter and length were analyzed. The influences of these factors on implant success and survival and on marginal bone loss (MBL) were evaluated. Success criteria were evaluated following The International Congress of Oral Implantologists (ICOI) Pisa Consensus implant health scale [16]:

- Absence of pain or tenderness upon function, palpation, or percussion
- Absence of clinical implant mobility in any direction with loads less than 500 g
- 2 mm radiographic bone loss from the initial surgery
- No history of exudate

The term “survival” refers to stable implants but with a history of or potential for clinical problems. The implant failure criteria involved the following conditions:

- Presence of pain on palpation, percussion, or function
- Horizontal and/or vertical mobility
- Radiographic bone loss > 1/2 the length of the implant
- Uncontrolled exudate
- No longer present intraorally

### Marginal bone loss (MBL) evaluation

Panoramic radiographs were taken after implant placement and during the follow-up visits of 3 months after implant placement, 3 months after functional loading, and 5 years after functional loading.

**Fig. 3:**
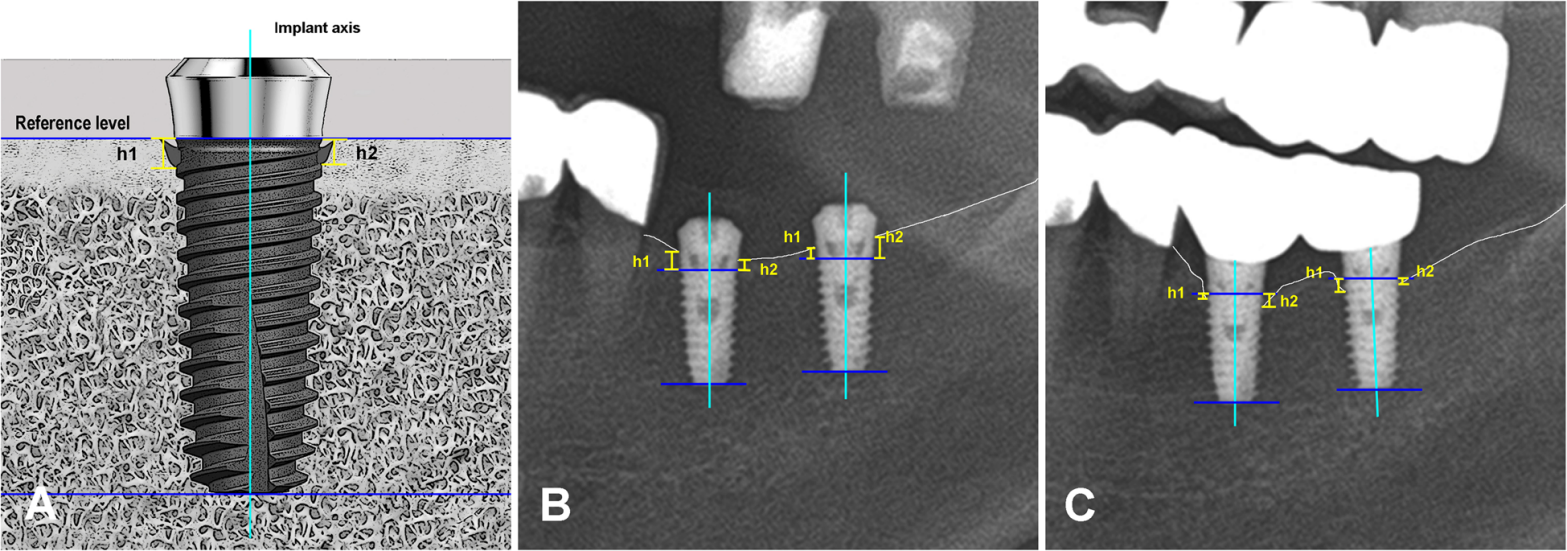
An example of the measurement of the MBL on the mesial and distal sides of the implant system (**a**) using panoramic radiographs taken after placement (**b**) and 1 year after loading (**c**). Marginal bone level on the mesial side, h1; marginal bone level on the distal side, h2. Both radiographs (**b**–**c**) depict the Stella® implant system 4 mm in diameter and 8.5 mm in length placed in the same patient

MBL was measured from the panoramic radiographs by measuring the distance from the implant platform to the coronal bone-to-implant contact point. The distance was measured on both mesial and distal sides three times each, and the mean was regarded as the representative value (Fig. 3a, b). The MBL was calculated as the difference of values between initial implant placement and 3 months after implant placement, 3 months after loading, and 5 years after loading. MBL was measured by the ImageJ® (National Institute of Mental Health, Bethesda, USA), a valid imaging software for peri-implant marginal bone measurements when the appropriate method is applied [17].

### Statistical analysis

The comparison of MBL around the implant at different time periods was analyzed by paired sample *t* tests using the SPSS 25 software® (Statistical Package for Social Sciences; SPSS, Inc., Chicago, USA). ANOVA testing was performed to identify statistical significance. Survival rates between the different implant diameter groups were evaluated, and *P* values < 0.05 were considered statistically significant.

## Results

One hundred five implants were installed in sixty-one patients. Two implants out of the 105 implants were removed due to implant failure. In one patient who had ablation maxillectomy surgery, we attempted to rehabilitate the upper first molar edentulous site with a 5.0-mm wide diameter and 7.0-mm long implant, but the implant was lost after 3 months following placement due to the lack of proper bone quality and quantity. In another patient with a similar poor medical condition, we rehabilitated the edentulous site using a socket lifting procedure, but because of the very small amount of remaining alveolar bone, the 5.0-mm wide diameter and 7.0-mm long implant was considered a failed implant and was removed after 33 months following placement.

Among the 61 patients, 28 were males and 33 were females. The average patient age at placement was 63.7 years with a range of 31 to 88 years. For the 105 total implants, implants with a diameter of 4.0 mm were placed 51 times, 4.5-mm wide implants were placed 41 times, and 5.0-mm wide implants were placed 13 times. The 4.0-mm and 4.5-mm diameter implants were used most frequently (49%, 42%) (Table 2). Regarding the length of the implants, 8.5-mm length implants were predominantly used 65 times, 10 mm implants were used 23 times, and 7 mm implants were used 17 times (Table 3). In terms of location, 73 implants were placed in the mandible, and 31 implants were placed in the maxilla. Only one implant was placed in the anterior maxilla (Table 4).

**Table 2:** Implant diameter and insertion distribution

Diameter of the implant	Number	Percentage
4.0 mm	51	48.6
4.5 mm	41	39
5.0 mm	13	12.4
Total	105

**Table 3:** Implant length and insertion distribution

Length of the implant	Number	Percentage
7 mm	17	16
8.5 mm	65	62
10 mm	23	22
Total	105

**Table 4:** Implant placement locations

Position	Maxilla	Mandible	Total
Anterior	1	0	1
Posterior	31	73	104
Total	32	73	105

### Success and survival rate

At the end of the 6-year observation period, the success rate of Stella® implants was 98.1%. Among the 105 implants placed in this study, 2 were considered to be failures. The survival rate for both the 4.0-mm diameter implants and 4.5-mm diameter implants was 100% and was 84.6% for the 5.0-mm diameter implants (Table 5). The survival rate results of the ANOVA one-way test showed statistically non-significant results (*p* > 0.05).

**Table 5:** Implant success and survival rates

Diameter	Success/failure (total)	Survival rate*
4.0	51/0 (51)	100
4.5	41/0 (41)	100
5.0	11/2 (13)	84.6
Total	103/105	98

*Survival rate results were not significant among the three diameter groups

### Marginal bone loss (MBL) evaluation

The MBL values were measured and calculated periodically. The mean MBL 3 months after placement was 0.25 ± 0.71 mm on the mesial side of the implant and 0.15 ± 0.69 mm on the distal side of the implant. The mean MBL was 0.67 ± 1.28 and 0.46 ± 0.90 mm at 3 months after functional loading, 0.85 ± 0.89 and 0.69 ± 0.99 mm at 1 year of functional loading, and 0.90 ± 0.61 and 0.74 ± 0.75 mm at 5 years of functional loading on the mesial and distal sides of the implant (Table 6). MBL at 3 months and at 5 years of loading differs significantly (*p* < 0.05).

**Table 6:** Marginal bone loss (MBL) evaluation on the mesial and distal sides of the implant according to the observation period

	Marginal bone resorption (mm) (mean ± SD)		*t* test *p* value*	
	Mesial	Distal	Mesial	Distal
3 months after placement	0.25 ± 0.71	0.15 ± 0.69	.033	.001
3 months after functional loading	0.67 ± 1.28	0.46 ± 0.90	.001	.001
1 year after functional loading	0.85 ± 0.89	0.69 ± 0.99	.022	.001
5 years after functional loading	0.90 ± 0.61	0.74 ± 0.75	.001	.001

*p values are based on the paired *t* test. The MBL at 3 months after placement, at 3 months loading, and at 1 year after loading and 5 years after loading differ significantly (*p <* 0.05)

## Discussion

Implant placement is now a routine procedure in the field of dentistry performed to replace missing teeth and relies on successful osseointegration, characterized by a biological strong link of the implant in bone tissue. This can be influenced by several external factors that disrupt the peri-implant microenvironment at the implant site or during the preparation phase of the site [18].

In this study, we report on 5-year follow-up results of a tapered, S&E treated implant system. This implant system demonstrated a survival rate of 98% (two were removed due to failure). The survival rates of 4.0 and 4.5-mm diameter implants were 100% and was 84.6% for 5.0-mm diameter implants. However, the survival rate results showed no statistical significance among the 4.0-, 4.5-, and 5.0-mm diameter implants. The results are in accordance with other studies where the failure rate was high in the maxilla and in the molar regions. This is affected by several factors such as the poor quality and quantity of the bone and the high degree of occlusal loading in the molar regions [19–21]. The success rate of this implant system during the 5-year follow-up period was 98.1%.

One of the failed implants cases belonged to a patient who had ablation maxillectomy surgery in the maxillary posterior region with insufficient bony volume. Three months after installation, the implants were found with mobility and were removed. The other failed implant belonged to a patient with hypertension and osteoporosis, where we performed a socket lifting procedure in the posterior maxilla. Three months after loading (33 months after placement), the implants were removed due to peri-implantitis (MBL of 2.71 mm). Both of the failed implants were a 5.0-mm diameter and 7-mm long implants that was placed in the maxillary posterior region. According to some studies, the use of wide-diameter (≥ 5 mm) implants with short implants did not show a significant influence on implant survival rates [22–24]. Similarly, a study by Javed et al. revealed that the role of implant diameter on the long-term survival of implants placed in the posterior maxilla [25]. On the other hand, the use of short dental implants < 8 mm (4–7 mm) is considered to be a more effective and simpler method for rehabilitating atrophic ridges compared to surgical techniques such as bone grafting or maxillary sinus grafting [22]. Although short dental implants show similar MBL, prosthetic failure, and complication rates to standard length implants, they should be used with caution because they present a greater risk of implant failure. Considering the increased failure risk of using short dental implants along with the individual patient-related risks, the length of the implant may be one of the influencing factors causing these implant failures.

According to a study by Albrektsson et al., 1 mm of peri-implant bone loss during the first year of function followed by vertical bone loss less than 0.2 mm annually after the implant’s first year of service is accepted as the criteria for implant success [26]. The quality and the quantity of bone surrounding the implant is considered to be one of the essential factors that influences the long-term success of implant treatment and is the decisive factor in the morphology, quality, and the esthetics of the soft tissue sealing the implant-supported restoration [17]. The evaluation of MBL after a period of more than 1 year after functional loading should be one of the indicators to predict the long-term success of the implant because the majority of implant failures occurs within this period of time [27]. In our study, the mean MBL at 3 months following implant placement was 0.25 ± 0.71 on the mesial side and 0.15 ± 0.69 on the distal side of the implant system. MBL 3 months following functional loading was 0.67 ± 1.28 and 0.46 ± 0.90 on the mesial and distal sides of the implant, respectively. MBL at 1 year following functional loading in our study was 0.85 ± 0.89 and 0.69 ± 0.99 on the mesial and distal aspects of the implants, respectively. At 5 years of functional loading, the mean MBL was 0.90 ± 0.61 and 0.74 ± 0.75 for the mesial and distal aspects of the implants, respectively.

Physiological bone remodeling around dental implants is a widely noted principle in the literature [28–31]. A comparative analyzing between the MBL changes in platform-switched bone level (BL) with the platform matched soft tissue level (TL) implant found that both implant systems had similar MBL and showed high clinical success rate [28]. Similarly, a study on the MBL changes between platform-switched implants with different mismatching distances reported that the increased horizontal mismatching did not have influenced on the MBL, but the implant-abutment height had more significant influence on the peri-implant preservation [29]. The reason of greater MBL values on the mesial aspects of the TL implants found in our study could be explained by the involvement of a machined collar, and the intraosseous positioning of the smooth-rough border of the implant causes greater bone loss in the region [30–32] and also by the inevitable mesial drifting of adjacent anterior teeth could make some more interdental space which should be cleaned by patient. Although the MBL changes were greater on the mesial aspects of the TL implants, the crestal bone remodeling basically occurs above the SLA treated surface; therefore, a sufficient bone support could be maintained.

In comparison, a retrospective clinical study by Kang et al., the average MBL around SLA treated implants was 0.09 ± 0.26 mm, 0.14 ± 0.41 mm, and 0.17 ± 0.45 mm at 5, 7, and 9 years, respectively [33]. Although our results show higher values of MBL at 5 years of functional loading, the values are within the threshold of implant success criteria [34]. The results of our study are in accordance with the implant success criteria proposed by Albrektsson et al. and The ICOI Pisa Consensus implant health scale. Based on the findings, the Stella® implant system exhibits successful clinical prognosis in the rehabilitation of edentulous patients [16, 26].

**Fig. 4:**
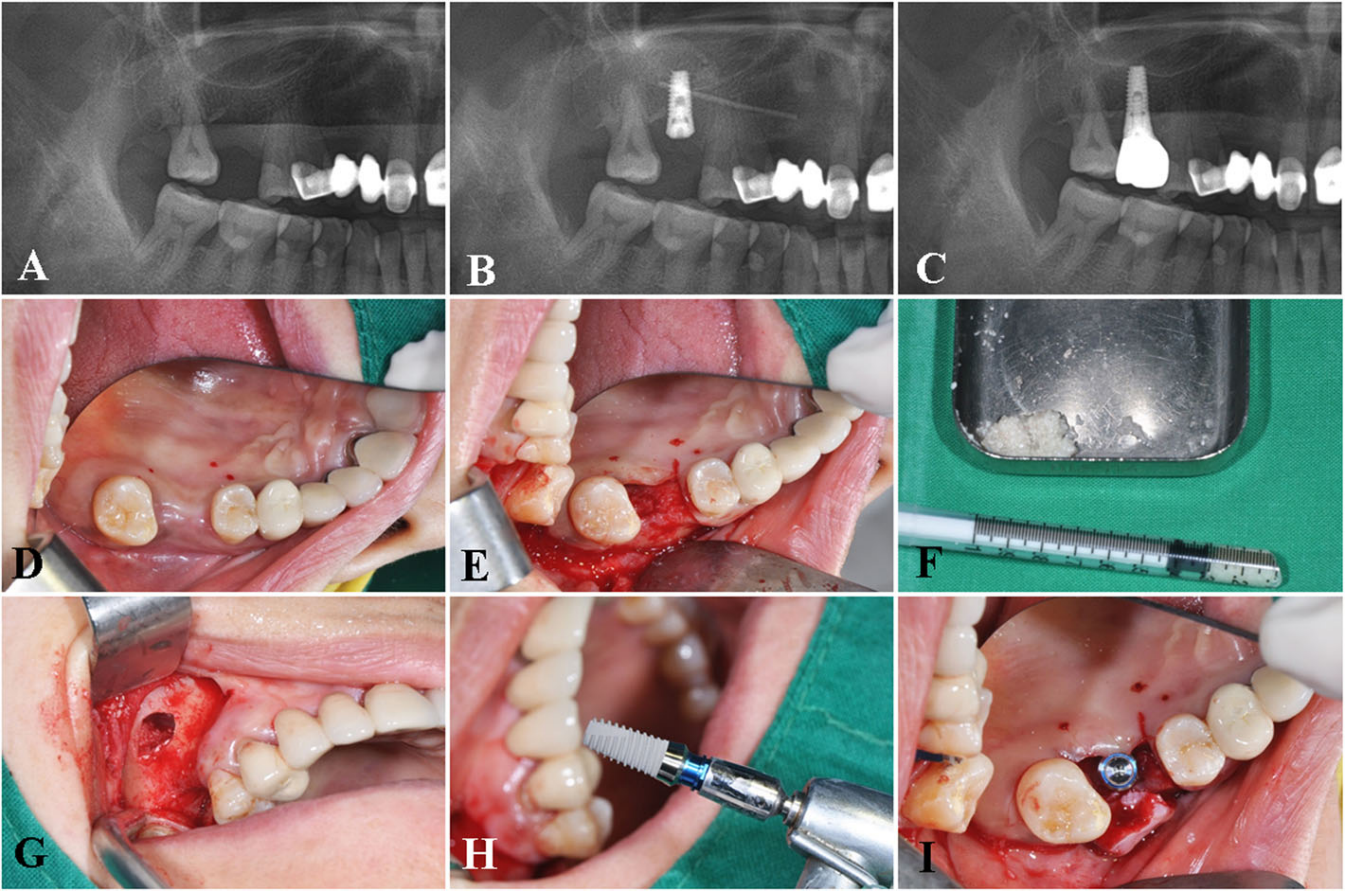
A case of a Stella® implant placed in combination with a sinus grafting procedure. The pre-operative (**a**), postoperative panoramic view (**b**), panoramic view after prosthesis delivery (**c**), intra-operative view of the sinus graft, and implant placement procedure using an allogenic particulate grafting material, OraGraft® (LifeNet Health Inc., VA, USA) (**d**–**j**)

**Fig. 5:**
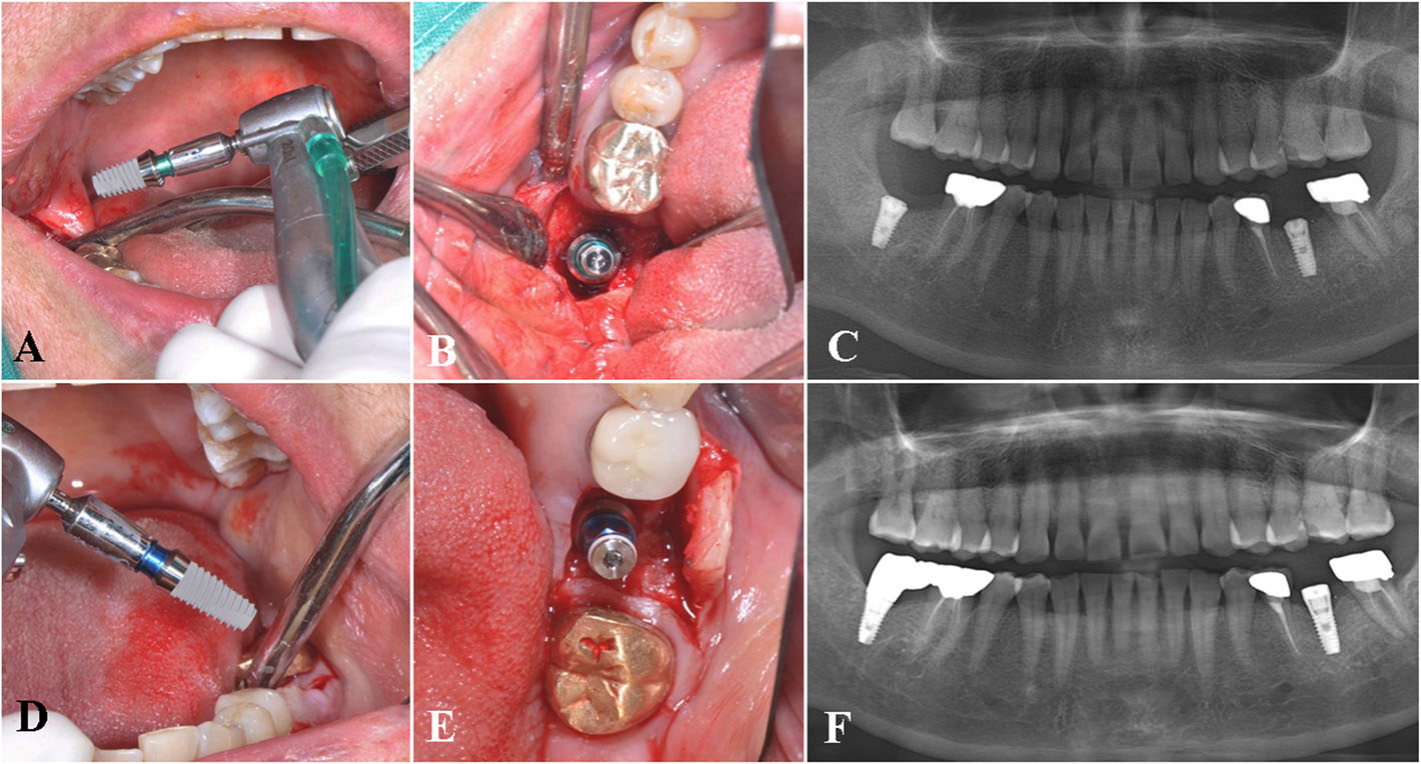
A representative case of bilateral implants placed in the posterior mandible. Intraoperative view of a 4.5 mm in diameter and 7 mm in length implant placed in the #47 area (**a**–**b**), postoperative panoramic view (**c**), placement of a 4.0 mm in diameter, 8.5 mm in length implant in the #36 area (**d**–**e**), and postoperative panoramic view (**f**)

**Fig. 6:**
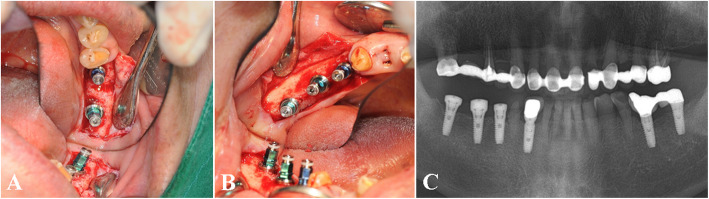
A case of multiple implants placed in the posterior mandible. Intraoperative view of the #36, 37 (**a**) and #45, 46, 47 implants (**b**), postoperative panoramic view of the multiple implants in the mandible (**c**)

The clinical feasibility and benefits of the Stella® implant system were also demonstrated in various clinical conditions where implant survival and success may be compromised, such as placement in the posterior maxilla with sinus grafting (Fig. 4a–j), bilateral implant placement in bone of poor quality (Fig. 5a–f), and multiple implants placed in the posterior mandible (Fig. 6a–c). According to several retrospective studies and a systematic review, augmentation procedures such as sinus floor elevation in the posterior maxilla are related to increased chances of early implant failures [35–38]. However, when practitioners assess the suitability of the patient for implant surgery and carefully select cases in the absence of systemic conditions, the implant system may exhibit a successful prognosis as seen in our study.

## Conclusion

According to our 5-year observation period, the success rate of Stella® implants was 98.1%, providing a satisfactory result. The MBL was in accordance with the success criteria of dental implant treatment. This implant system designed with of a tapered, S&E, and TL exhibited great performance in a diverse clinical situation that results in predictable and successful treatment outcomes.

## Data Availability

The datasets used and/or analyzed during the current study are available from the corresponding author on reasonable request.
